# [P_4_H]^+^[Al(OTeF_5_)_4_]^–^: protonation of white phosphorus with the Brønsted superacid H[Al(OTeF_5_)_4_]_(solv)_†
†Dedicated to Prof. Dr Peter Jutzi on the occasion of his 80^th^ birthday.
‡
‡Electronic supplementary information (ESI) available. See DOI: 10.1039/c8sc03023e


**DOI:** 10.1039/c8sc03023e

**Published:** 2018-08-23

**Authors:** Anja Wiesner, Simon Steinhauer, Helmut Beckers, Christian Müller, Sebastian Riedel

**Affiliations:** a Institut für Chemie und Biochemie , Freie Universität Berlin , Fabeckstr. 34/36 , 14195 Berlin , Germany . Email: c.mueller@fu-berlin.de ; Email: s.riedel@fu-berlin.de

## Abstract

A sustainable transformation of white phosphorus (P_4_) into chemicals of higher value is one of the key aspects in modern phosphorus research. Even though the chemistry of P_4_ has been investigated for many decades, its chemical reactivity towards the simplest electrophile, the proton, is still virtually unknown. Based on quantum-chemical predictions, we report for the first time the successful protonation of P_4_ by the Brønsted acid H[Al(OTeF_5_)_4_]_(solv)_. Our spectroscopic results are in agreement with acid-mediated activation of P_4_ under protonation of an edge of the P_4_-tetrahedron and formation of a three-center two-electron P–H–P bond. These investigations are of fundamental interest as they permit the activation of P_4_ with the simplest electrophile as a new prototype reaction for this molecule.

## Abstract 2

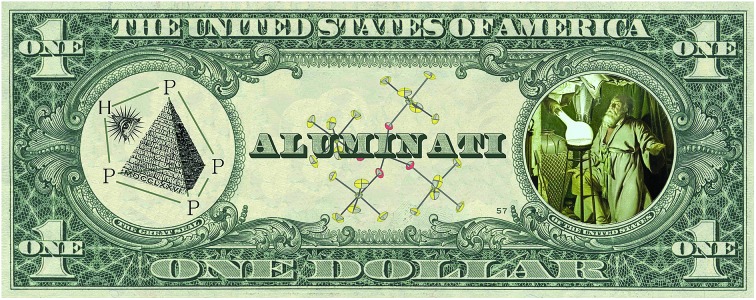
The structure of protonated white phosphorus in solution has been revealed for the first time.

## Introduction

White phosphorus (P_4_), discovered by Henning Brand in 1669 while searching for the philosopher's stone, is the thermodynamically least stable and most reactive form of phosphorus at room temperature and consists of tetrahedral P_4_ molecules. Despite its spontaneous flammability and severe toxicity, P_4_ is the easiest form to produce on an industrial scale and is therefore the commercially most important allotrope.^1^ Especially its conversion to PCl_3_ is of high interest, as it is a base chemical for the production of many organophosphorus compounds.

**Fig. 1:**
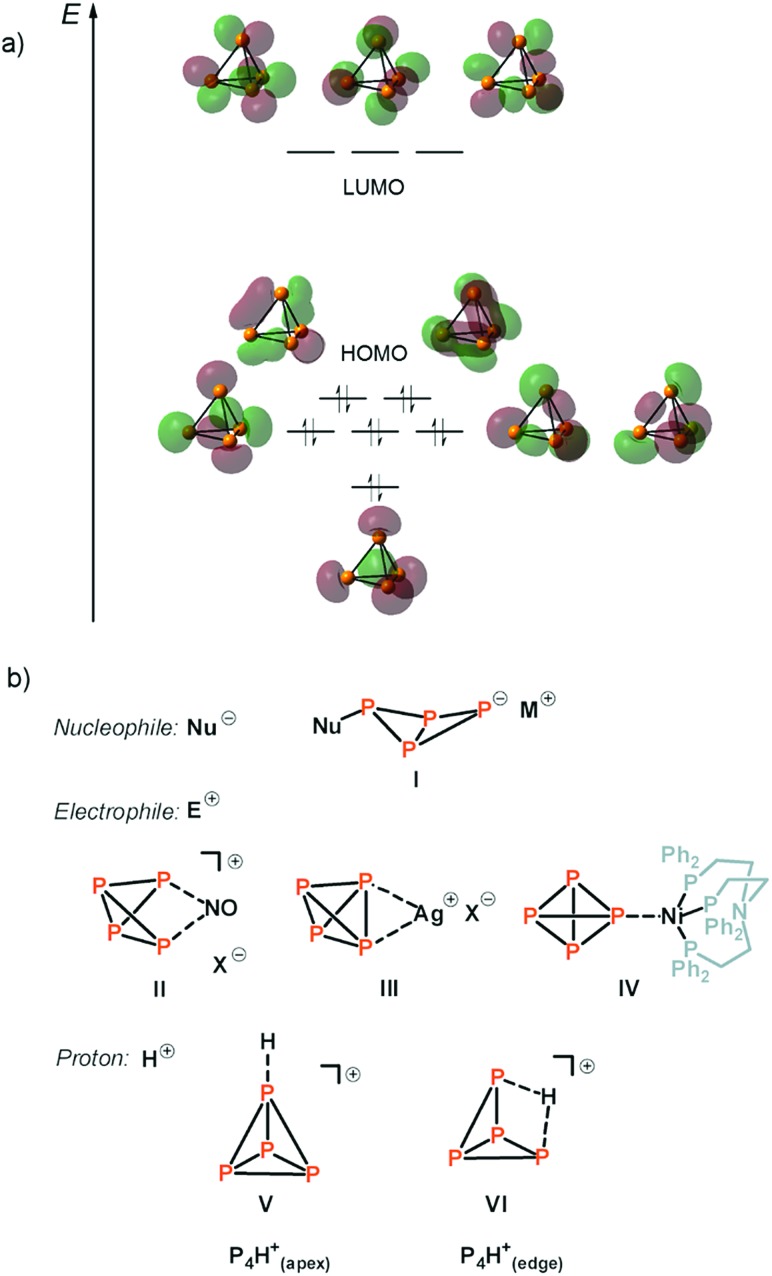
Molecular orbital scheme of P_4_ (a)^8^ and examples of nucleophilic and electrophilic attacks at P_4_ and the assumed structures for protonated P_4_ (b).

From a historical point of view, two important chemical reactions of P_4_ are described in every good textbook of inorganic chemistry:^2^ (a) the slow oxidation of P_4_ vapor to P_4_O_10_ under emission of light. This chemoluminescence has coined the name phosphorus, which is derived from the greek mythology (“light-bearer”). (b) the activation and disproportionation of P_4_ by aqueous solutions of alkali metal hydroxides. In this way, the industrially relevant phosphine gas (PH_3_) is obtained in high purity next to the alkali metal salt of hypophosphorous acid (NaH_2_PO_2_). More recent studies deal with the degradation of white phosphorus in the presence of other strong nucleophiles, such as organolithium and organomagnesium compounds, carbenes or silylenes, under the topic “P_4_-activation and functionalization”.^3^–^6^ From a mechanistic point of view, a charged nucleophile (Nu^–^) interacts with one of the three energetically degenerate LUMOs of the P_4_ molecule (Fig. 1a) under opening of the P_4_ tetrahedron to yield a substituted butterfly-like bicyclo[1,1,0]tetraphospha-butane anion (Fig. 1b, **I**).^7^

Electrophiles, on the other hand, should react at an edge of the tetrahedron, as the two energetically degenerate highest occupied molecular orbitals (HOMO and HOMO–1) have large coefficients at two adjacent phosphorus atoms (Fig. 1a). The situation is, however, much more complicated and the formation of various products is usually observed in this seemingly simple reaction. In the case of Ph_2_P^+^ and NO^+^, the insertion of these small molecules into one of the P–P bonds is indeed observed, as also theoretically predicted for NO^+^ (Fig. 1b, **II**).^9^–^11^ In the case of Ag^+^ as an example of an electrophilic transition metal center, a weak coordination of Ag^+^ to the edge of the P_4_ tetrahedron occurs (Fig. 1b, **III**).^12^ However, depending on the steric demand of the metal fragment, the coordination of P_4_*via* the apex can be enforced, even though the interaction of the energetically low-lying HOMO–5 with the electrophile is necessary to achieve this coordination mode (Fig. 1b, **IV**).^13^

In contrast to the experimental observations made for the reaction of P_4_ with nucleophiles as well as coordinatively and electronically unsaturated transition metal complexes, experimental proof for the structure of the elusive [P_4_H]^+^ cation in solution is still missing in the literature. In fact, weak acids do not react with P_4_ due to the rather poor nucleophilicity and weak basicity of white phosphorus. Common strong acids, such as sulfuric acid (H_2_SO_4_) and nitric acid (HNO_3_) cannot be used for the generation of [P_4_H]^+^ as they directly oxidize P_4_ to either phosphorous acid (H_3_PO_3_) and sulfur dioxide (SO_2_), or to phosphoric acid (H_3_PO_4_), nitrogen oxide (NO_2_) and water (H_2_O), respectively. Hydrogen chloride (HCl) can react with P_4_ to form phosphine gas (PH_3_) and phosphorus trichloride (PCl_3_). Based on *ab initio* calculations, Fluck *et al.*^14^ predicted in 1979 that the weakly bound proton in [P_4_H]^+^ is located at the apex of the tetrahedron (Fig. 1b, **V**), while protonation at the edge was predicted to be energetically less favored (Fig. 1b, **VI**). The authors exclude protonation at the P_3_-face. More recent *ab initio* molecular orbital calculations at the MP2/6-31G(d,p) level of theory in 1996 by Abboud, Yáñez and co-workers reveal, however, that the thermodynamically most favourable process is the protonation at the edge under formation of a three-center two-electron (3c-2e) P–H–P bond (Fig. 1b, **VI**).^15^ The same group determined the gas-phase basicity of P_4_ by means of Fourier transform ion cyclotron resonance mass spectrometry. In 2000, Ponec and co-workers provided an additional theoretical support for the existence of a non-classical 3c-2e P–H–P bond in [P_4_H]^+^ using the generalized population analysis.^16^ More recently, Lobayan and Bochicchio used a topological analysis of the electron density to describe the 3c-2e P–H–P bond in [P_4_H]^+^.^17^

## Results

Taking the above-mentioned considerations into account, we anticipated that strong acids of conjugated weakly coordinating and non-reactive anions should be excellent reagents for the protonation of P_4_. Reed and Nixon, for instance, have shown that phosphabenzenes can be protonated by the *in situ* generated Brønsted superacid H(CHB_11_Me_5_Br_6_).^18^ Also these phosphorus heterocycles are known for their extremely weak basicity. As one of us^19^ has recently reported a novel aluminum-based superacidic system containing the weakly coordinating anion [Al(OTeF_5_)_4_]^–^, we report here now the synthesis and the first spectroscopic proof on the structure of [P_4_H]^+^ in solution.

According to quantum-chemical calculations at the B3LYP/def2-TZVPP level, the protonation of P_4_ can be achieved by a medium consisting of the Brønsted superacid H[Al(OTeF_5_)_4_]_(solv)_ and *ortho*-difluorobenzene (*o*-DFB), see eqn (1).^19^ This is due to a slightly lower proton affinity of *o*-DFB (741.6 kJ mol^–1^) compared to P_4_ (748.4 kJ mol^–1^), as computed at CCSD(T)/aug-cc-pVTZ level of theory.1
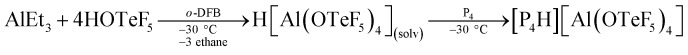



**Fig. 2:**
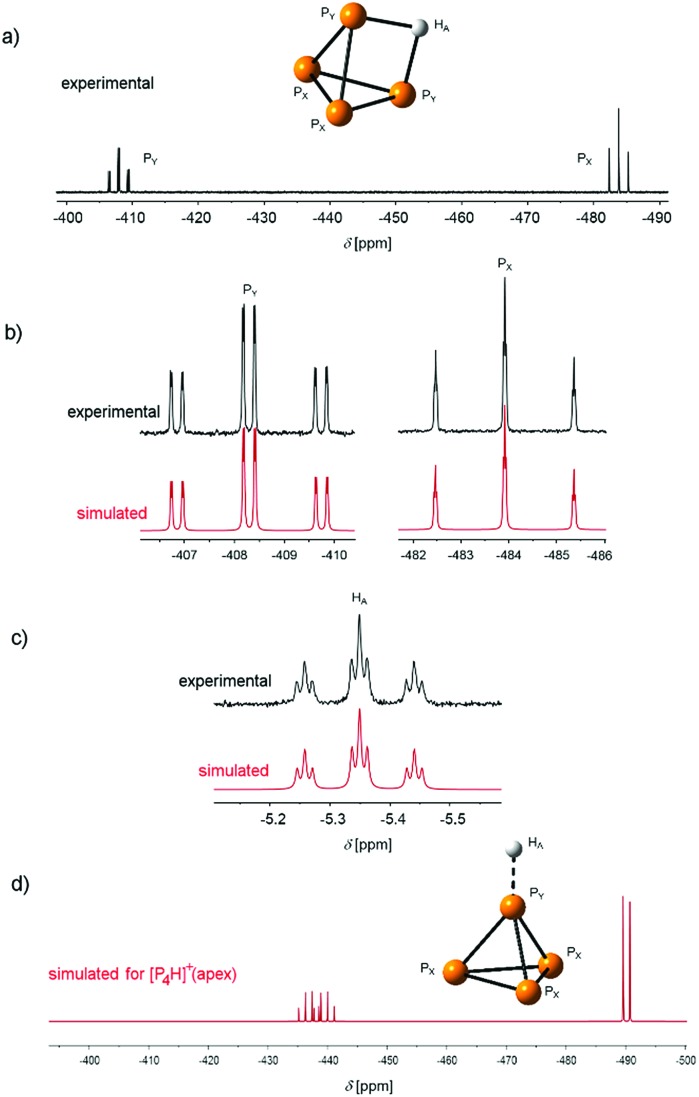
(a) Low-temperature (*T* = –40 °C) experimental ^31^P NMR spectra of [P_4_H][Al(OTeF_5_)_4_] in *o*-DFB (external lock: [D_6_]acetone). The atom labelling is indicated in accordance with the AX_2_Y_2_ spin system. (b) ^31^P NMR spectrum (162 MHz, top) and simulated P_X_ and P_Y_ signals (bottom) of the species [P_4_H]+(edge). (c) Experimental and simulated ^1^H NMR spectrum (401 MHz). The full spectra are provided in Fig. S1 and S2.‡ (d) Simulated ^31^P NMR P_X_ and P_Y_ signals of the species [P_4_H]+(apex).

The reaction product of P_4_ and the Brønsted superacid was obtained as a temperature-, moisture- and oxygen-sensitive salt. It shows a clean low-temperature proton-coupled ^31^P NMR spectrum with two equally intense signals at *δ* = –481.7 and *δ* = –405.8 ppm with a weak roof effect (Fig. 2a). No other signals were observed in the ^31^P NMR spectrum in the region between *δ* = 400 ppm and *δ* = –800 ppm.

This spectrum, which also reveals an additional splitting owing to the higher order of the system, is in accordance with an AX_2_Y_2_ spin system (^1^*J*(^31^P_X_, ^31^P_Y_) = 233.95 Hz, ^1^*J*(^1^H_A_, ^31^P_Y_) = 36.70 Hz, ^2^*J*(^31^P_X_, ^1^H_A_) = 4.91 Hz). This can only result from the protonation of the P_4_ molecule at the P–P-edge. Furthermore, a triplet of triplets at *δ* = –5.35 ppm appears in the ^1^H NMR spectrum (Fig. 2c) showing the corresponding couplings of the proton to P_X_ and P_Y_, respectively. Interestingly, both the chemical shifts and coupling constants are in excellent agreement with the simulated spectra of [P_4_H]+(edge), obtained by quantum-chemical calculations (Fig. 2b and c and S3, Table S1‡). For comparison reasons, Fig. 2d shows the simulated ^31^P NMR spectrum of the species [P_4_H]+(apex), which clearly differs from the experimental results. The NMR studies clearly prove the presence of [P_4_H][Al(OTeF_5_)_4_] and that P_4_ is protonated at an edge of the tetrahedron as predicted by quantum-chemical calculations.^15^–^17^

**Fig. 3:**
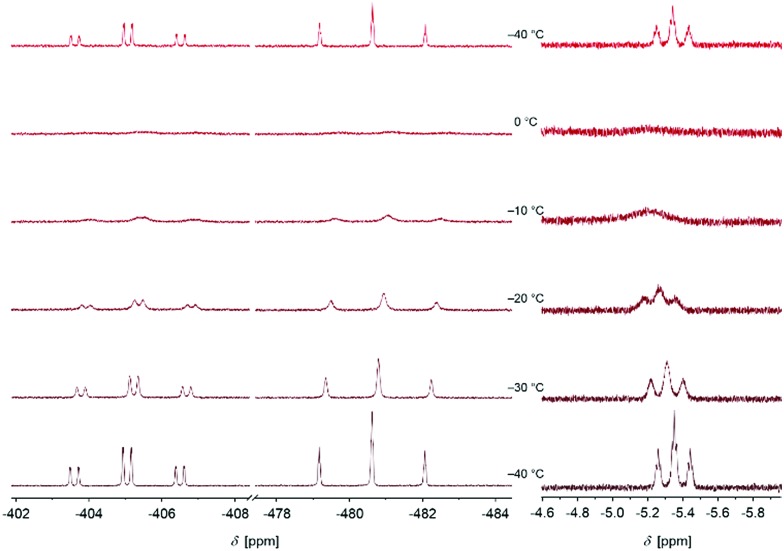
Excerpt of the ^31^P and ^1^H NMR spectra of [P_4_H][Al(OTeF_5_)_4_] in *o*-DFB (external lock: [D_6_]acetone) at various temperatures. The sample was first measured at *T* = –40 °C, annealed stepwise to *T* = 0 °C and cooled down again to *T* = –40 °C afterwards.

We further started to investigate the dynamics of the cation in solution. Interestingly, variable temperature NMR spectroscopy indicates a coalescence of the signals at *T* = –10 °C (Fig. 3).

The triplet of triplets observed at *T* = –40 °C in the ^1^H NMR spectrum broadens with increasing temperature resulting in a broad singlet (approx. FWHM = 75 Hz) at the coalescence temperature. The chemical shift slightly changes from *δ* = –5.35 ppm at *T* = –40 °C to *δ* = –5.19 ppm at *T* = 0 °C. In the ^31^P NMR spectrum, a similar process is observed. The two signals are broadened at the coalescence temperature and shifted to a higher field by 0.5 ppm for P_X_ and P_Y_ at *T* = 0 °C. This process is reversible by re-cooling the sample to *T* = –40 °C again. No signal for P_4_ is detected during this process. Based on these observations, we anticipate a dynamic intramolecular migration of the proton on the P_4_ surface. From the experimental dynamic-NMR data, the corresponding barrier can be estimated to Δ*G*^‡^ = 54.2 kJ mol^–1^.

We noticed, that if an excess of P_4_ is present in the reaction mixture, the previously described [P_9_]^+^ cation^20^ is formed next to [P_4_H]^+^. Upon warming a sample containing a mixture of [P_4_H]^+^ and P_4_ from *T* = –40 °C to *T* = –10 °C, a fast and full conversion to [P_9_]^+^ is observed, as detected by NMR spectroscopy. This observation indicates that activation of the P_4_ molecule by protonation already occurs at low temperature, while broad band UV/Vis irradiation is necessary to form [P_9_]^+^ from a P_4_/[P_4_NO]^+^ mixture, as reported in the literature before.^10^

The [P_4_H]^+^ cation was further analyzed by means of mass spectrometry. In the mass spectrum (positive mode), a signal allocated to [P_4_H]^+^ appears at *m*/*z* = 124.9. In addition, signals due to [P_4_]^+^ (*m*/*z* = 123.8) as well as of [^*n*^Te]^+^ and [^*n*^TeH]^+^ in a natural isotope distribution (*n* = 122, 124–126) arise with less intensity. Furthermore, the cations [P_3_]^+^, [*o*-DFB]^+^, [*o*-DFB–H]^+^ and [P_5_]^+^ were found. The mass spectrum recorded in the negative mode shows only signals of the four anions [AlF_3_(OTeF_5_)]^–^, [AlF_2_(OTeF_5_)_2_]^–^, [AlF(OTeF_5_)_3_]^–^ and [Al(OTeF_5_)_4_]^–^, see Fig. S7–S9.‡

**Fig. 4:**
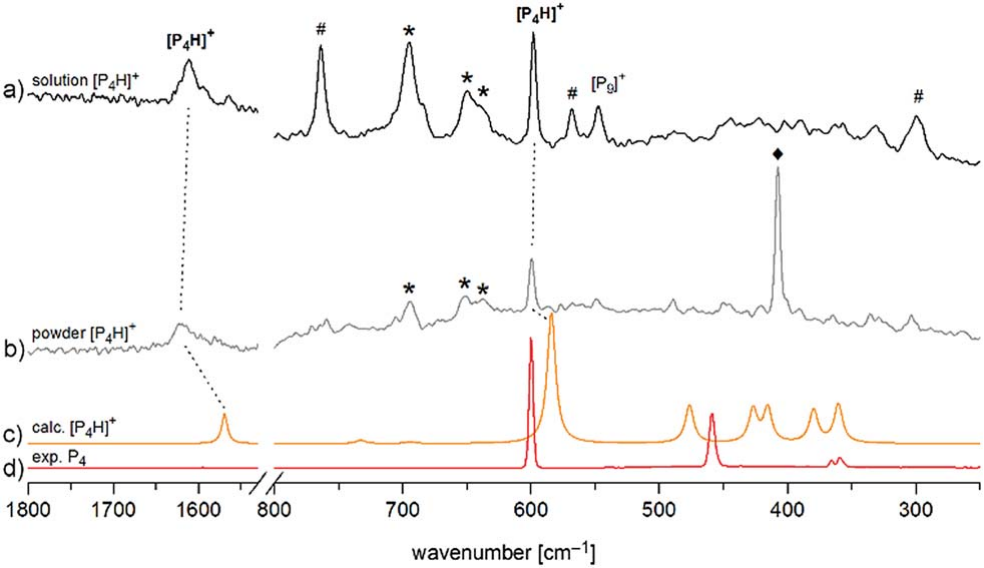
Enlarged Raman spectrum of (a) [P_4_H][Al(OTeF_5_)_4_] in *o*-DFB at *T* = –30 °C and (b) [P_4_H][Al(OTeF_5_)_4_] washed with *n*-pentane at *T* = –78 °C, (c) calculated spectrum of [P_4_H]^+^ at the B3LYP/def2-TZVPP level of theory and (d) experimental spectrum of solid P_4_ at *T* = –196 °C. Bands of the anion [Al(OTeF_5_)_4_]^–^ at *ν̃* = 695, 650 and 637 cm^–1^ are marked by an asterisk (*). Bands of the solvent (*o*-DFB: #, *n*-pentane: ♦) are indicated as well. Full spectra are provided in Fig. S5 and S6.‡

Finally, we investigated [P_4_H][Al(OTeF_5_)_4_] by means of Raman spectroscopy both in an *o*-DFB solution at *T* = –30 °C and as a neat powder at *T* = –78 °C in the solid state (Fig. 4). In both spectra, two prominent bands can be observed. The band around *ν̃* = 1615 cm^–1^ corresponds to the symmetrical P–H–P stretching mode and the band at *ν̃* = 598 cm^–1^ occurs slightly shifted with respect to the breathing mode of neat P_4_ and is assigned to the corresponding mode of [P_4_H]^+^.

Both the experimental band positions agree well with the computed wavenumbers at the B3LYP/def2-TZVPP level of theory at *ν̃* = 1569 cm^–1^ (A_1_) and *ν̃* = 584 cm^–1^ (A_1_), respectively. Further Raman bands are predicted between *ν̃* = 360 cm^–1^ and *ν̃* = 476 cm^–1^ but they are difficult to assign in the experimental spectrum due to their rather low intensities and their partial interference with the bands of [P_9_]^+^ impurities. It should be pointed out that special care must be taken by isolating [P_4_H][Al(OTeF_5_)_4_] as a solid, as one sample exploded during the Raman measurement at dry ice temperature after approx. 300 scans at 75 mW, Fig. 4b. Attempts to record low-temperature IR spectra of the [P_4_H]^+^ cation were unsuccessful, as the strongest IR band of [P_4_H]^+^ is hidden by very prominent bands of the anion at *ν̃* = 713 cm^–1^ and *ν̃* = 695 cm^–1^. Also, the strongest band of *o*-DFB occurs at *ν̃* = 750 cm^–1^. Nevertheless, low temperature (*T* = –30 °C) IR spectra of the liquid phase of [P_4_H][Al(OTeF_5_)_4_] have been recorded using a glass fiber ATR head, which are provided in Fig. S10 and S11.‡

**Fig. 5:**
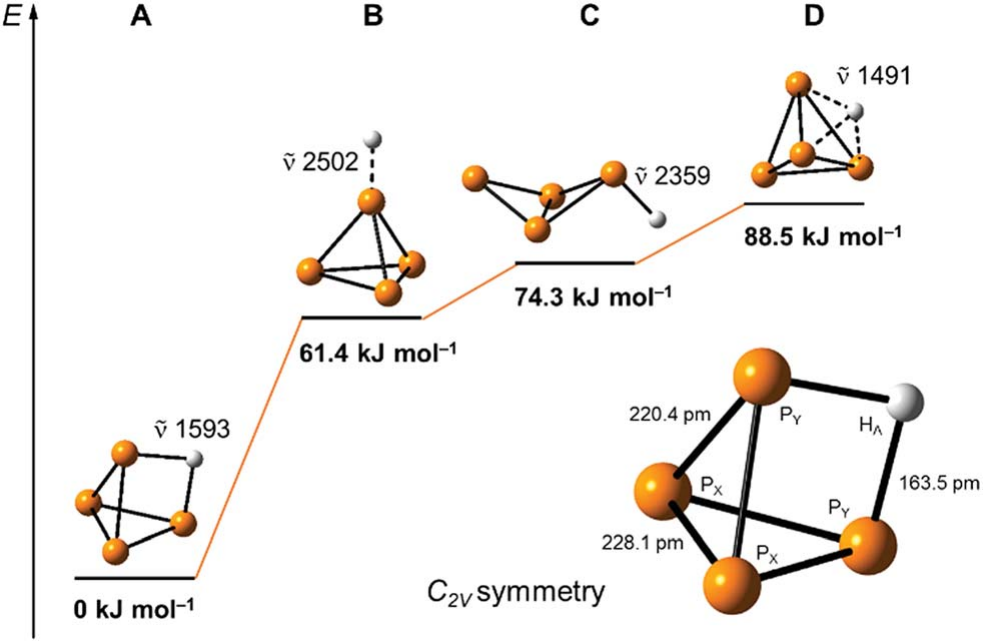
Computed relative energies and P–H vibrations of optimized [P_4_H]^+^ structures at the CCSD(T)/aug-cc-pVTZ level of theory.

Our quantum-chemical calculations at the coupled-cluster CCSD(T)/aug-cc-pVTZ level agree very well with the experimental results of the protonation of a P_4_ edge. This position is also expected from the MO diagram, where the HOMO orbital is located along the P_4_ edge (Fig. 1a), leading to a three-center two-electron P–H–P bond. This gives rise to a *C*_2v_ symmetric structure with an elongation of the P_Y_···P_Y_ bond of 20.4 pm compared to the bond length of 221.8 pm in the P_4_ tetrahedral structure. The bond distances P_Y_–P_X_ and P_X_–P_X_ are less affected by 1.4 and 6.3 pm, respectively (Fig. 5). The computed minimum structure for protonation at the apex of the P_4_ molecule is 61.4 kJ mol^–1^ higher in energy than the global minimum structure. An apex protonation would also lead to a computed P–H stretching mode at *ν̃* = 2502 cm^–1^, which is more than *ν̃* = 900 cm^–1^ above the experimentally observed band at *ν̃* = 620 cm^–1^. Surprisingly, the protonation and simultaneous opening of the tetrahedral structure lead to a P_4_-butterfly type minimum structure (Fig. 5), while protonation of the triangle surface shows a higher order saddle point. Both structures will be higher in energy by 74.3 and 88.5 kJ mol^–1^ compared to the global minimum structure of [P_4_H]^+^, see Fig. 5. For details of the computed structural parameters see Tables S2 and S3.‡

## Conclusions

Based on these results, we could reveal for the first time the structure of protonated white phosphorus in solution. Both experimental results and quantum-chemical calculations provide evidence for a protonation at the edge of the P_4_ molecule. The opening of the P_4_-tetrahedron *via* the simplest electrophile (H^+^) under formation of a three-center two-electron P–H–P bond is of fundamental interest for understanding the reactivity of this intriguing phosphorus allotrope. It is expected that this groundbreaking result is important for the development of chemical processes related to the activation and further functionalization of elemental phosphorus by electrophiles.

## Conflicts of interest

There are no conflicts to declare.

## Supplementary Material

Supplementary information
