# Supplementary material for: [P4H]+[Al(OTeF5)4]–: protonation of white phosphorus with the Brønsted superacid H[Al(OTeF5)4](solv)
Source: Chem Sci. 2018 Aug 23;9(36):7169–73. doi: 10.1039/c8sc03023e (PMC6151472; doi:10.1039/c8sc03023e)
Supplement: Supplementary file 1 [file SC-009-C8SC03023E-s001.pdf]

Electronic Supplementary Material (ESI) for Chemical Science.
This journal is © The Royal Society of Chemistry 2018

Supplementary Materials

$[P_4H]^+[Al(OTeF_5)_4]^-$: Protonation of White Phosphorus with the Brønsted Superacid $H[Al(OTeF_5)_4]_{(solv)}$

Anja Wiesner, Simon Steinhauer, Helmut Beckers, Christian Müller*, Sebastian Riedel*

Freie Universität Berlin, Institut für Chemie und Biochemie, Anorganische Chemie, Fabeckstr. 34/36, 14195 Berlin, Germany.

*Correspondence to: c.mueller@fu-berlin.de, s.riedel@fu-berlin.de.

Experimental Details:

Caution! White phosphorus is toxic and extremely pyrophoric. Special safety precautions have to be considered!

All preparative work was carried out using standard Schlenk techniques. Glass ware was greased with Triboflon III. The pentafluoro-orthotelluric acid was prepared as described elsewhere.[1] Triethylaluminium ($AlEt_3$, 93 %) was handled inside a glove box with an atmosphere of dry argon (O_2 < 0.5 ppm, H_2O < 0.5 ppm). All solvents were freshly dried with CaH_2 before use. P_4 was purified as followed: a piece of white phosphorus (~ 150 mg) was added to a beaker filled with degassed H_2O (~ 50 mL) and melted using a heating gun. The molten P_4 was collected with a Pasteur pipette and dropped into fresh H_2O, in which it solidified spontaneously. This procedure was repeated twice. Subsequently, the molten P_4 was again collected with a Pasteur pipette and dropped into dry THF. The solvent (THF) was removed via syringe and the remaining P_4 was dried quickly in vacuo and stored in the glove-box. Raman spectra were recorded on a Bruker MultiRAM II equipped with a low-temperature Ge detector (1064 nm, 50 mW, resolution 2 cm^{-1}). IR spectra of cooled samples were measured on a Bruker Vertex70 FTIR spectrometer equipped with a matrix unit connected with a glass fiber ATR attachment (resolution 1 cm^{-1}). NMR spectra were recorded on a JEOL 400 MHz ECS or ECZ spectrometer with a [D6]acetone capillary (external lock) with trichlorofluoromethane $CFCl_3$, tetramethylsilane (TMS), trimethyl phosphate (TMP) as standards. Chemical shifts and couplings constants are given as simulated by Mestrenova 10.0.[2] Mass spectra were collected on a WATERS Synapt G2-S HDMS spectrometer from a solution of the compound in dry *o*-DFB.

Caution! Protonated white phosphorus is temperature sensitive and tends to explode if it is isolated as solid or warmed up to room temperature in solution.

Quantum-chemical Details: Molecular structures of all phosphorus containing compounds in their electronic ground states were optimized at the CCSD(T) level. Correlation consistent triple-ζ all-electron basis sets (aug-cc-pVTZ) were used for the structure optimizations. These calculations were carried out with the MOLPRO program for coupled-cluster calculations.[3]
In addition, the Turbomole program[4] was used to perform calculations at the unrestricted Kohn-Sham DFT level, using the B3LYP hybrid functional[5-7] in conjunction with the valence triple-ζ basis set with two sets of polarization functions (def2-TZVPP).[8]

Minima on potential energy surfaces were characterized by normal mode analysis. Thermochemical data is provided without counterpoise correction, but includes zero-point energy corrections as obtained from harmonic vibrational frequencies.
NMR chemical shifts and spin-spin couplings been computed using GAUSSIAN 09W[9] at B3LYP/aug-cc-pVTZ-J[10] level with Gauge-Independent Atomic Orbital (GIAO) method.[11-15]

Preparation of $[P_4H][Al(OTeF_5)_4]$: In a two necked Schlenk flask equipped with a stop-cock 47 mg (0.41 mmol) $AlEt_3$ were dissolved in 5 mL *ortho*-difluorobenzene. The solution was cooled with liquid nitrogen and degassed. 395 mg (1.65 mmol) $HOTeF_5$ were condensed onto the solution. A bubbler was connected to the flask and the reaction mixture was warmed up to –30 °C, resulting in a yellow solution. 50 mg (0.40 mmol) P_4 was dissolved in cold *n*-pentane (−40 °C) and added via syringe. The reaction mixture was stirred for 1 h at −40 °C. A color change to orange was observed.

Isolation of protonated white phosphorus was performed by condensing *n*-pentane onto the solution of $[P_4H][Al(OTeF_5)_4]$ in *o*-DFB. A dense ionic phase is formed covered by an organic phase (*o*-DFB and *n*-pentane) which can be removed via syringe. The remaining solvent in the ionic phase was removed in vacuo. The product was obtained as a red solid.

^{1}H NMR (401 MHz, *o*-DFB, *n*-pentane, ext. [D6]acetone, −40°C):
δ=7.0 (m, *o*-DFB), 4.6 (d, TMP), 3.0 (sex, acetone), 1.3 (m, *n*-pentane), 0.9 (m, *n*-pentane), −5.4 (tt, $1H_A$, $^1J(^1H,^{31}P_Y)$=36.7 Hz, $^2J(^1H,^{31}P_X)$=4.9 Hz, $[P_4H]^+$);

^{19}F NMR (377 MHz, *o*-DFB, *n*-pentane, ext. [D6]acetone, −40°C):
δ=–38.6 (m, $1F_A$, $^2J(^{19}F,^{19}F)$=188 Hz, $^1J(^{125}Te,^{19}F_A)$=3402 Hz, $[Al(OTeF_5)_4]^-$), –45.5 ppm (m, $4F_B$, $^1J(^{125}Te,^{19}F_B)$=3506 Hz));

^{31}P NMR (162 MHz, *o*-DFB, *n*-pentane, ext. [D6]acetone, −40°C):
δ=–408.3 (m, $2P_X$, $^1J(^{31}P_X,^{31}P_Y)$=234.0 Hz), −483.9 ppm (m, $2P_Y$);

^{27}Al NMR (104 MHz, *o*-DFB, *n*-pentane, ext. [D6]acetone, −40°C):
δ=48.0 ppm (s, FWHM=550 Hz, $[Al(OTeF_5)_4]^-$);

FT-Raman (−40°C): $\tilde{\nu}$=3086$^{\#}$ (w), 2935$^{\blacklozenge}$ (m), 2918$^{\blacklozenge}$ (w), 2910$^{\blacklozenge}$ (w), 2905$^{\blacklozenge}$ (w), 2894$^{\blacklozenge}$ (w), 2877$^{\blacklozenge}$ (m), 2863$^{\blacklozenge}$ (m), 1610 (s, ν(P-H)), 1270$^{\#}$ (m), 1026$^{\#}$ (s), 764$^{\#}$ (s), 695 (s, ν(O-Te-F)), 650 (m, ν(Te-F)), 641 (sh, ν(Te-F)), 598 (s, $\nu(P_4H)_{breath}$), 568$^{\#}$ (w), 547$^{\bullet}$ (w), 444 (w), 422 (w), 415$^{\bullet}$ (w), 402 (w), 389$^{\bullet}$ (w), 363$^{\bullet}$ (w), 357 (w), 330 (w), 300$^{\#}$ (m), 198$^{\#}$ (w), 68 (vs) $^{\blacklozenge}$ denote *n*-pentane, $^{\#}$ denote *o*-DFB, $^{\bullet}$ denote $[P_9]^+$;

ESI-MS (4 kV): *m/z* (%) positive: 124.903 (39) $[P_4H]^+$, negative: 225 (1) $[TeF_5^-]$, 322.9 (0.5) $[AlF_3(OTeF_5)]^-$, 546.8 (1.1) $[AlF_2(OTeF_5)_2]^-$, 762.6 (14.7) $[FAl(OTeF_5)_3^-]$, 982.5 (100) $[Al(OTeF_5)_4^-]$.

Sample preparation for ESI-MS: The protonated white phosphorus in *o*-DFB was kept at −40 °C before the measurement. The cold solution was immediately injected to the mass spectrometer by a Hamilton syringe.

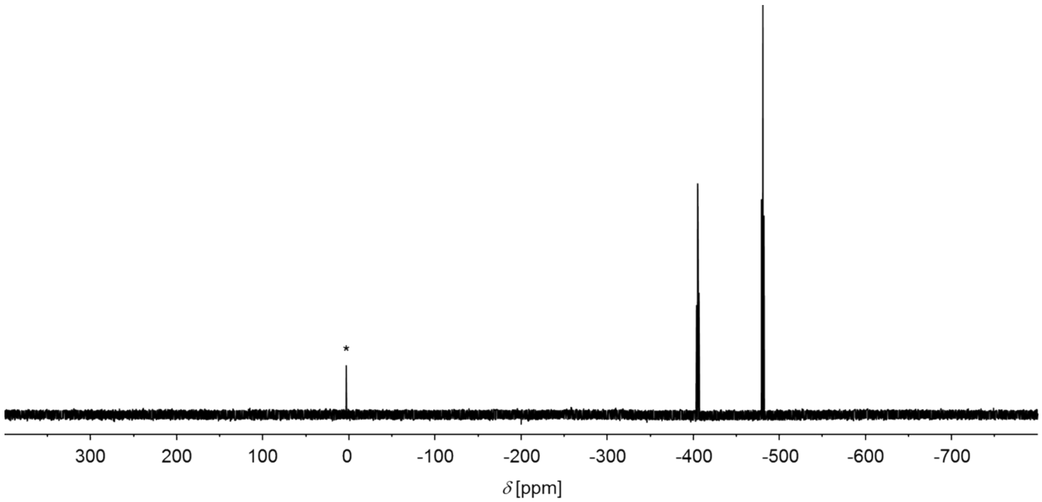

Fig. S1. Experimental ^{31}P NMR spectrum of $[P_4H][Al(OTeF_5)_4]$ in *o*-DFB (162 MHz, [D6]acetone (external lock), –40 °C) The standard trimethyl phosphate TMP is marked with a star (*).

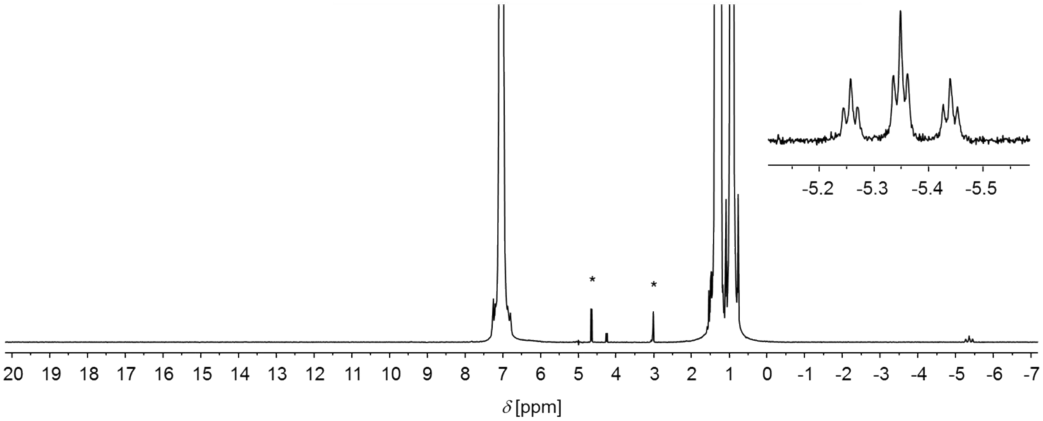

Fig. S2. Experimental ^{1}H NMR spectrum of $[P_4H][Al(OTeF_5)_4]$ in *o*-DFB (401 MHz, [D6]acetone (external lock), –40 °C) The external lock and the standard trimethyl phosphate TMP are marked with a star. Cut peaks are due to the solvents *o*-DFB and *n*-pentane at 0.9, 1.3 and 7 ppm.

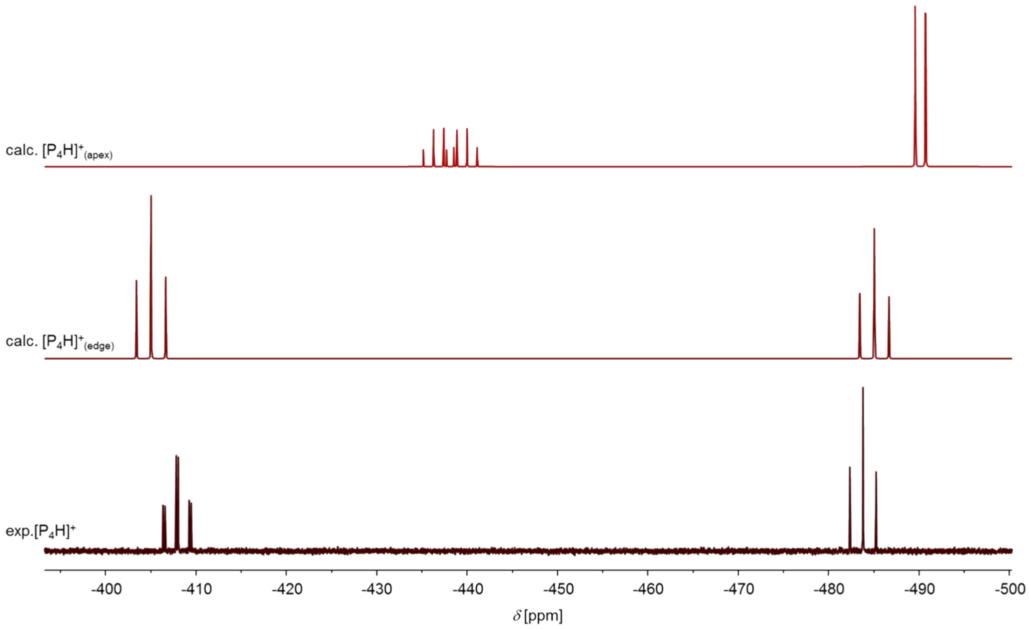

Fig. S3. Quantum-chemically calculated ^{31}P NMR spectra of the $[P_4H]^+$ moiety for the protonation on the apex (top) and edge (middle) compared to the experimental ^{31}P NMR spectrum (bottom) of $[P_4H][Al(OTeF_5)_4]$ in *o*-DFB (162 MHz, [D6]acetone (external lock), –40 °C). The GIAO calculations were performed with a B3LYP functional and an aug-cc-pVTZ-J basis set optimized for coupling constants.

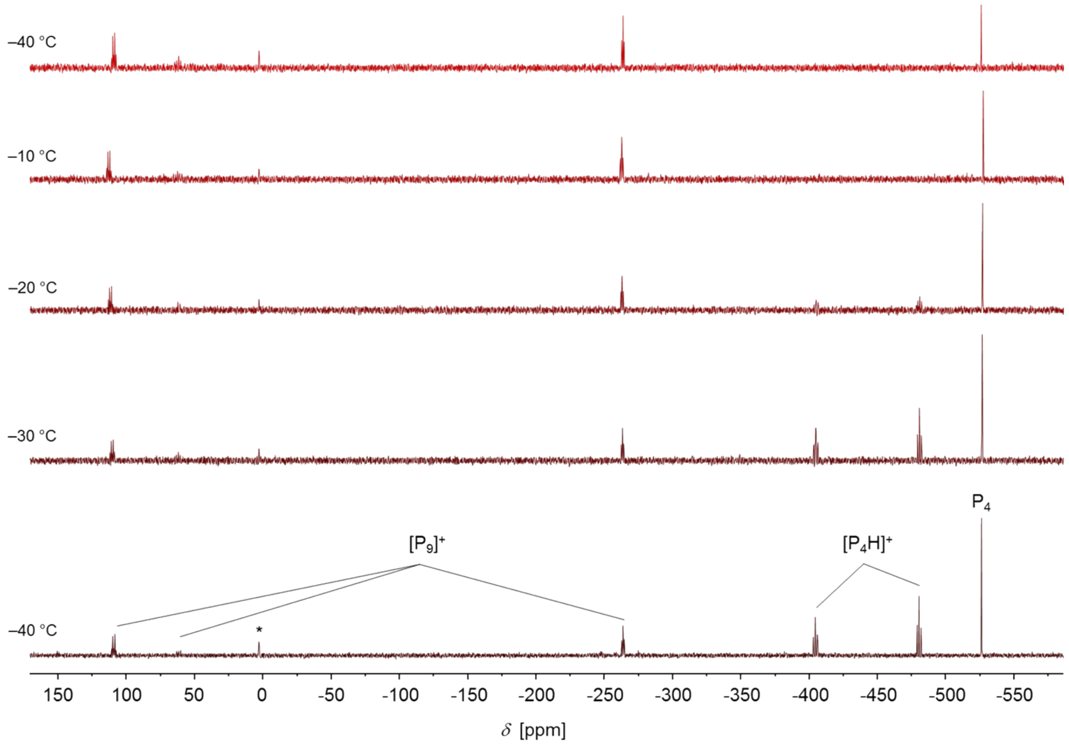

Fig. S4. Experimental ^{31}P NMR spectra of $[P_4H][Al(OTeF_5)_4]$ with an excess of P_4 in *o*-DFB (162 MHz, [D6]acetone (external lock)) at various temperatures. The sample was first measured at –40 °C, annealed stepwise to –10 °C and cooled to –40 °C afterwards. The standard trimethyl phosphate TMP is marked with a star (*).

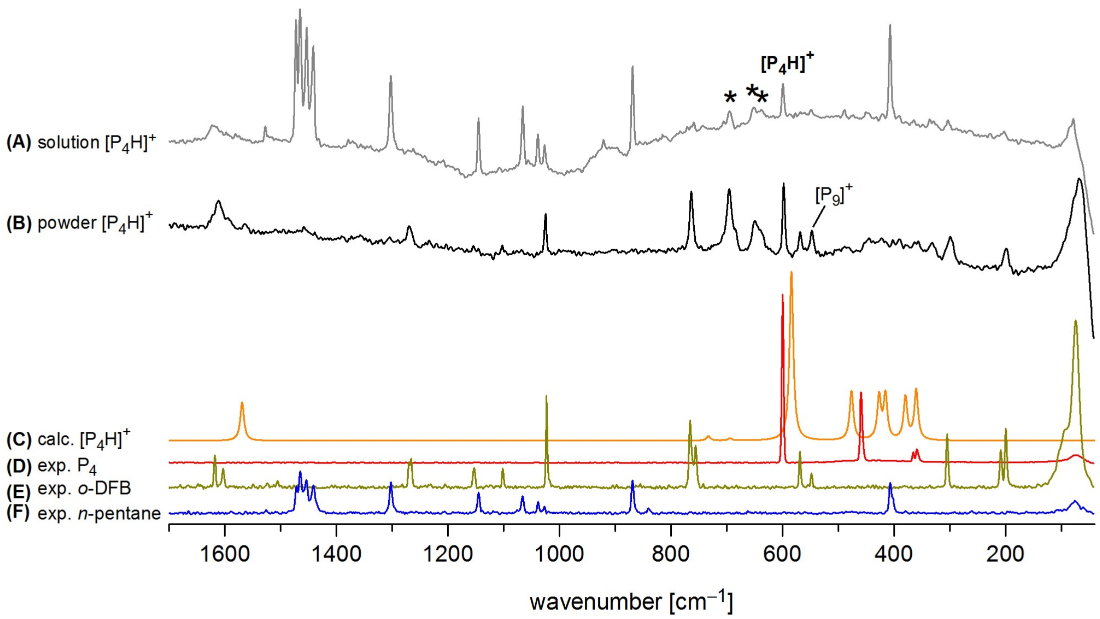

Fig. S5. Enlarged Raman spectrum of (A) $[P_4H][Al(OTeF_5)_4]$ in *o*-DFB at –30 °C, (B) $[P_4H][Al(OTeF_5)_4]$ washed with *n*-pentane at –196 °C, (C) calculated spectrum $[P_4H]^+$ at B3LYP/def2-TZVPP level of theory, (D) experimental spectrum of solid P_4 at –196 °C, (E) experimental spectrum of *o*-DFB at –196 °C and (F) experimental spectrum of *n*-pentane at –196 °C. Bands of the anion $[Al(OTeF_5)_4]^-$ at 695, 650 and 637 cm^{-1} are marked with a star (*).

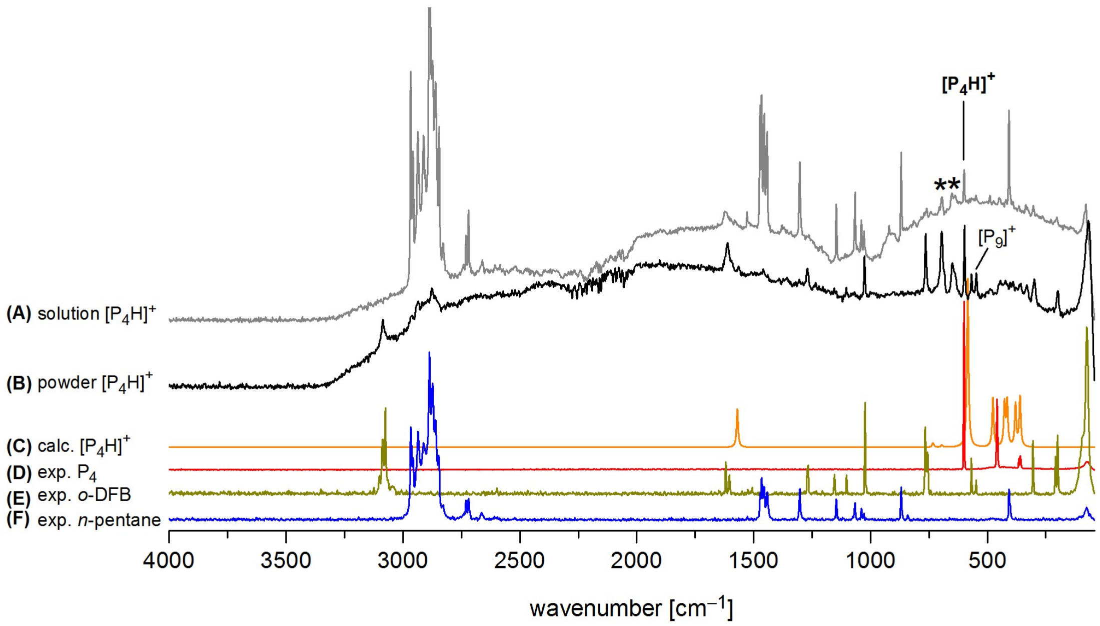

Fig. S6. Raman spectrum of (A) $[P_4H][Al(OTeF_5)_4]$ in *o*-DFB at –30 °C, (B) $[P_4H][Al(OTeF_5)_4]$ washed with *n*-pentane at –196 °C, (C) calculated spectrum $[P_4H]^+$ at B3LYP/def2-TZVPP level of theory, (D) experimental spectrum of solid P_4 at –196 °C, (E) experimental spectrum of *o*-DFB at –196 °C and (F) experimental spectrum of *n*-pentane at –196 °C (F). Bands of the anion $[Al(OTeF_5)_4]^-$ at 695, 650 and 637 cm^{-1} are marked with a star (*).

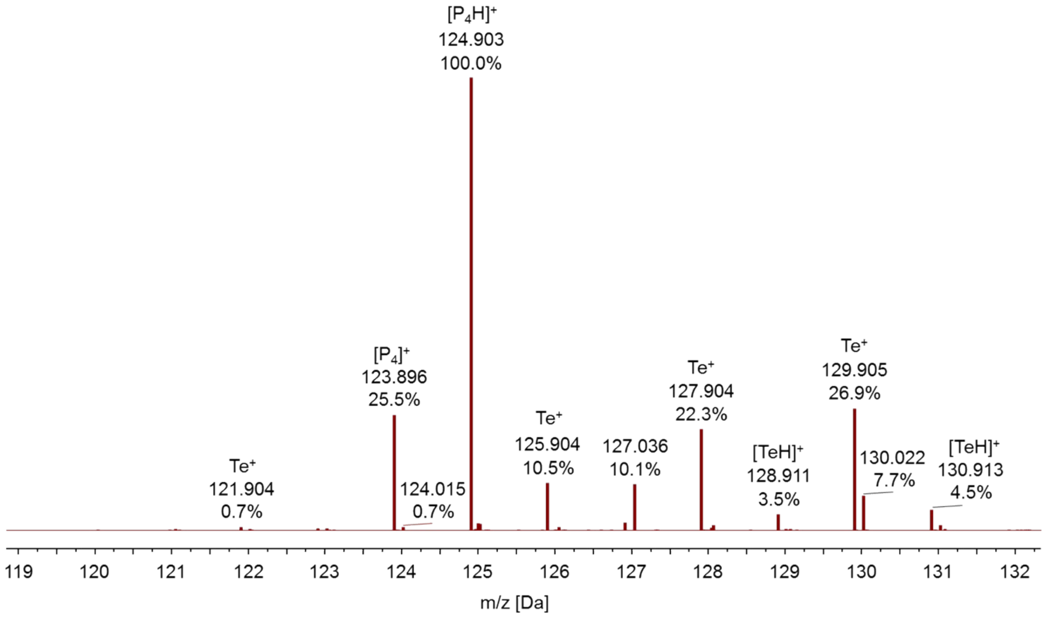

Fig. S7. Enlarged mass spectrum (positive mode) of $[P_4H][Al(OTeF_5)_4]$ in *o*-DFB.

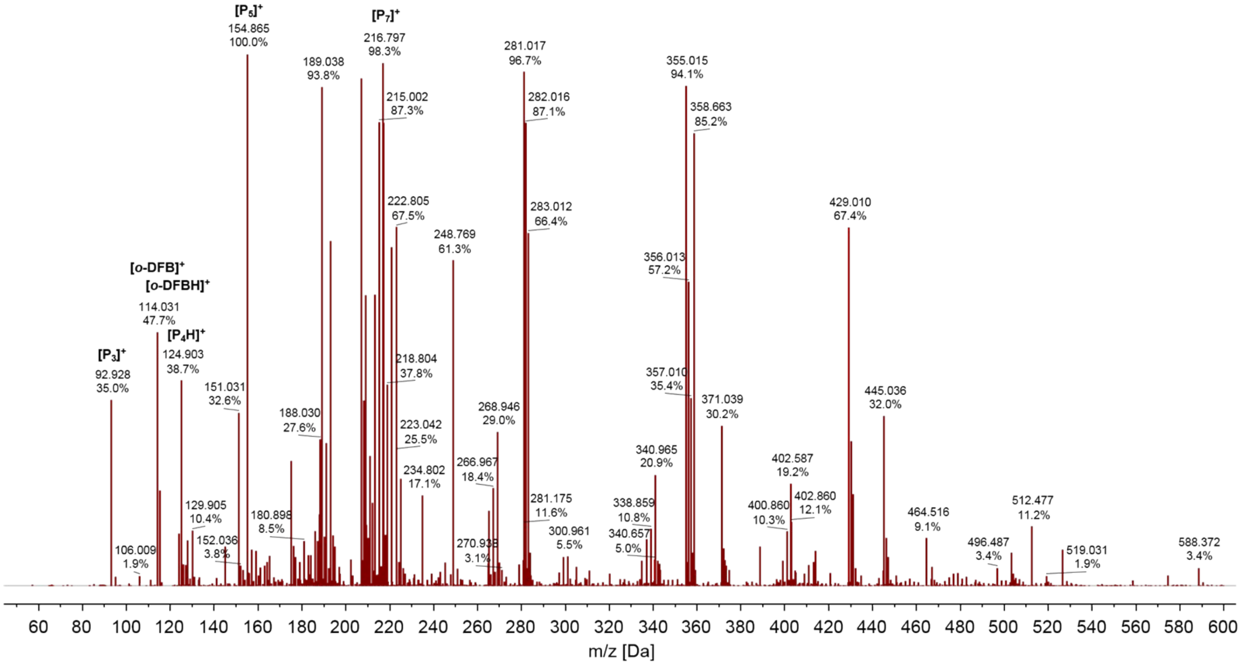

Fig. S8. Mass spectrum (positive mode) of $[P_4H][Al(OTeF_5)_4]$ in *o*-DFB.

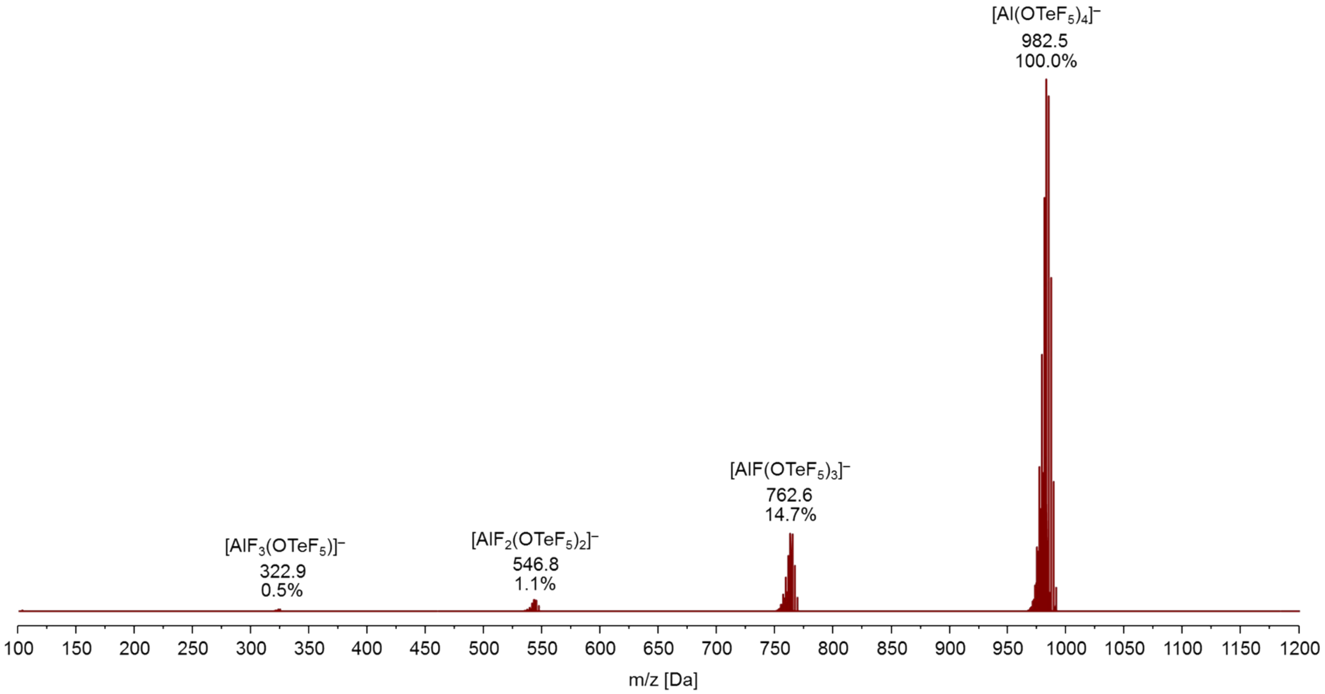

Fig. S9. Mass spectrum (negative mode) of $[P_4H][Al(OTeF_5)_4]$ in *o*-DFB.

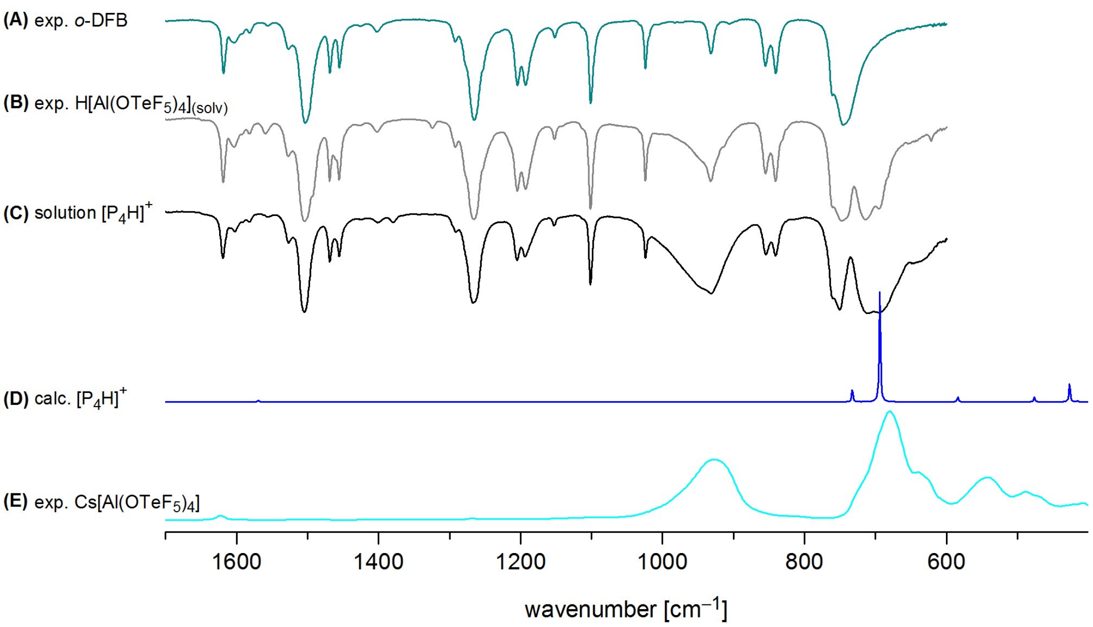

Fig. S10. Enlarged infrared spectrum of (**A**) *o*-DFB, (**B**) $H[Al(OTeF_5)_4]_{(solv)}$ in *o*-DFB, (**C**) $[P_4H][Al(OTeF_5)_4]$ washed with *n*-pentane, (**D**) calculated spectrum $[P_4H]^+$ at B3LYP/def2-TZVPP level of theory and (**E**) experimental spectrum of literature known solid $Cs[Al(OTeF_5)_4]{\cdot}CH_2Cl_2$ for comparison. Spectra A-C were measured at −30 °C.

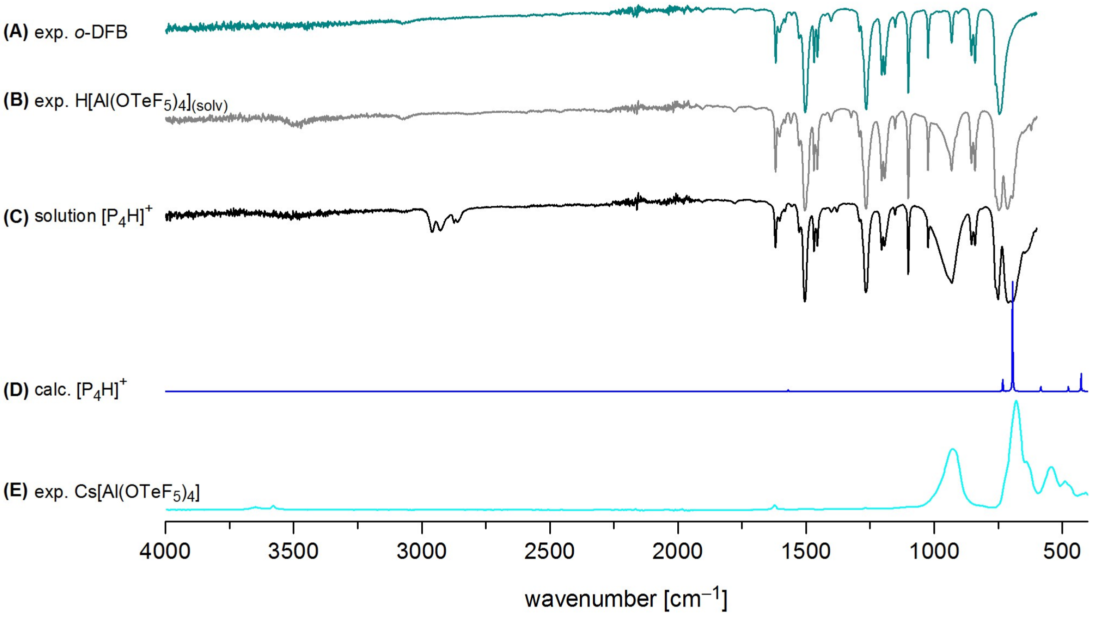

Fig. S11. Infrared spectrum of (**A**) *o*-DFB, (**B**) $H[Al(OTeF_5)_4]_{(solv)}$ in *o*-DFB, (**C**) $[P_4H][Al(OTeF_5)_4]$ washed with *n*-pentane, (**D**) calculated spectrum $[P_4H]^+$ at B3LYP/def2-TZVPP level of theory and (**E**) experimental spectrum of literature known solid $Cs[Al(OTeF_5)_4]{\cdot}CH_2Cl_2$ for comparison. Spectra A-C were measured at −30 °C.

Table S.1. Comparison of the experimentally obtained NMR parameter of $[P_4H]^+$ with computed chemical shifts (δ [ppm]) and coupling constants (J [Hz]) for P_4 as an anchor point, $[P_4H]^+_{(apex)}$ and $[P_4H]^+_{(edge)}$ at the DFT B3LYP/ aug-cc-pVTZ-J level of theory.

structure	assignment	number of nuclei	δ [ppm]*	$^2J(^1H,^{31}P_X)$ [Hz]	$^1J(^1H,^{31}P_Y)$ [Hz]	$^1J(^{31}P_X,^{31}P_Y)$ [Hz]	referred to exp. δ [ppm]
P_4 (T_D)	P	4	849.25	–	–	–	–529.7
$[P_4H]^+_{(apex)}$ (C_{3V})	P_X	1	809.69	8.49	417.86	182.49	–490.1
	P_Y	3	757.61	–		182.49	–438.1
$[P_4H]^+_{(edge)}$ (C_{2V})	P_X	2	804.58	7.41	–	261.15	–485.0
	P_Y	2	724.57	–	6.41	261.15	–405.0
			exp. δ [ppm]	exp. $^2J(^1H,^{31}P_X)$ [Hz]	exp. $^1J(^1H,^{31}P_Y)$ [Hz]	exp. $^1J(^{31}P_X,^{31}P_Y)$ [Hz]	
$[P_4H]^+_{(exp.)}$	H_A	1	–5.35	4.91	36.70	–	–
	P_X	2	–408.3	4.91	36.70	233.95	–
	P_Y	2	–483.9	–	–	233.95	–

*absolute computed shielding

Table S2. Comparison of the calculated bond length and the P−H−P angle of $[P_4H]^+$ at B3LYP/def2-TZVPP and CCSD(T)/aug-cc-pVTZ level of theory.

	$[P_4H]^+_{(edge)}$	$[P_4H]^+_{(edge)}$	$[P_4H]^+_{(apex)}$	$[P_4H]^+_{(biphenoidal)}$	$[P_4H]^+_{(triangle)}$
	**CCSD(T)/aug-cc-pVTZ**	**B3LYP/def2-TZVPP**	**CCSD(T)/aug-cc-pVTZ**	**CCSD(T)/aug-cc-pVTZ**	**CCSD(T)/aug-cc-pVTZ**
**bond**	**length [pm]**	**length [pm]**	**length [pm]**	**length [pm]**	**length [pm]**
P_X−P_X	228.137	226.777	230.106	247.481	–
P_X−P_Y	220.371	219.224	214.124	222.638	219.067
P_Y−P_Y	241.357	240.734	–	–	235.588
P_Y−H_A	163.528	163.573	140.254	142.807	172.706
P_X−P_Z	–	–	–	213.400	–
**angle**	∠ [°]	∠ [°]	∠ [°]	∠ [°]	∠ [°]
∠($P_YH_AP_Y$)	95.117°	94.760°	–	–	86.009°

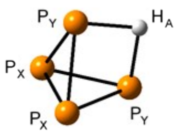
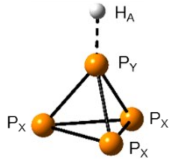
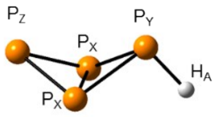
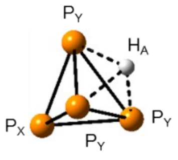

Table S 3. Calculated vibrational spectrum of $[P_4H]^+$ at CCSD(T)/aug-cc-pVTZ and B3LYP/def2-TZVPP level of theory.

**vibrational mode**	**CCSD(T)/aug-cc-pVTZ**	**B3LYP-/def2-TZVPP**	**B3LYP-/def2-TZVPP**	**B3LYP-/def2-TZVPP**
	**freq. [cm^{-1}]**	**freq. [cm^{-1}]**	**IR intensities [%]**	**Raman intensities [%]**
P_X–P_X stretch (A_1)	358.30	360.42	0	31
P_4 deform. (A_2)	381.46	379.66	0	27
P_Y–P_Y stretch (A_1)	428.40	426.82	0	30
P_2H rocking (B_2)	433.42	415.46	17	29
P_4 deform. (B_1)	476.63	476.37	5	29
P_4 breathing (A_1)	587.14	584.07	4	100
P_2H wagging (B_1)	711.69	732.67	100	2
asym. P_2H stretch (B_2)	749.18	693.98	12	3
sym. P_2H stretch (A_1)	1593.20	1569.17	1	23

References and Notes

1. K. Seppelt, D. Nothe, *Inorg. Chem.*, 1973, **12**, 2727.
2. C. Cobas *et al.*, *MestReNova* (Mestrelab Research S.L., 2015).
3. H.-J. Werner *et al.*, *MOLPRO, version 2015.1, a package of ab initio programs* (2015).
4. TURBOMOLE GmbH, *TURBOMOLE V7.0.1* (a development of University of Karlsruhe and Forschungszentrum Karlsruhe GmbH, 1989–2015).
5. A. D. Becke, *Phys. Rev. A*. 1988, **38**, 3098.
6. C. Lee, W. Yang, R. G. Parr, *Phys. Rev. B*., 1988, **37**, 785.
7. S. H. Vosko, L. Wilk, M. Nusair, *Can. J. Phys.*, 1980, **58**, 1200.
8. F. Weigend, R. Ahlrichs, *Phys. Chem. Chem. Phys.*, 2005, **7**, 3297.
9. M. J. Frisch, G. W. Trucks, H. B. Schlegel, G. E. Scuseria, M. A. Robb, J. R. Cheeseman, G. Scalmani, V. Barone, B. Mennucci, G. A. Petersson, H. Nakatsuji, M. Caricato, X. Li, H. P. Hratchian, A. F. Izmaylov, J. Bloino, G. Zheng, J. L. Sonnenberg, M. Hada, M. Ehara, K. Toyota, R. Fukuda, J. Hasegawa, M. Ishida, T. Nakajima, Y. Honda, O. Kitao, H. Nakai, T. Vreven, J. A. Montgomery, Jr., J. E. Peralta, F. Ogliaro, M. Bearpark, J. J. Heyd, E. Brothers, K. N. Kudin, V. N. Staroverov, R. Kobayashi, J. Normand, K. Raghavachari, A. Rendell, J. C. Burant, S. S. Iyengar, J. Tomasi, M. Cossi, N. Rega, J. M. Millam, M. Klene, J. E. Knox, J. B. Cross, V. Bakken, C. Adamo, J. Jaramillo, R. Gomperts, R. E. Stratmann, O. Yazyev, A. J. Austin, R. Cammi, C. Pomelli, J. W. Ochterski, R. L. Martin, K. Morokuma, V. G. Zakrzewski, G. A. Voth, P. Salvador, J. J. Dannenberg, S. Dapprich, A. D. Daniels, O. Farkas, J. B. Foresman, J. V. Ortiz, J. Cioslowski, D. J. Fox, *Gaussian 09* (Gaussian, Inc., Wallingford CT, 2009).
10. T. Enevoldsen, J. Oddershede, S. P. A. Sauer, *Theor. Chem. Acc.*, 1998, **100**, 275.
11. F. London, *J. Phys. Radium*, 1937, **8**, 397.
12. R. McWeeny, *Phys. Rev.*, 1962, **126**, 1028.
13. R. Ditchfield, *Mol. Phys.*, 1974, **27**, 789.
14. K. Wolinski, J. F. Hinton, P. Pulay, *J. Am. Chem. Soc.*, 1990, **112**, 8251.
15. J. R. Cheeseman, G. W. Trucks, T. A. Keith, M. J. Frisch, *J. Chem. Phys.*, 1966, **104**, 5497.
